# Signaling pathways governing the behaviors of leukemia stem cells

**DOI:** 10.1016/j.gendis.2023.01.008

**Published:** 2023-03-23

**Authors:** Shirin Azizidoost, Ava Nasrolahi, Mohadeseh Sheykhi-Sabzehpoush, Amir Anbiyaiee, Seyed Esmaeil Khoshnam, Maryam Farzaneh, Shahab Uddin

**Affiliations:** a Atherosclerosis Research Center, Ahvaz Jundishapur University of Medical Sciences, Ahvaz 6193673111, Iran; b Infectious Ophthalmologic Research Center, Ahvaz Jundishapur University of Medical Sciences, Ahvaz 6193673111, Iran; c Department of Laboratory, Imam Khomeini Hospital Complex, Tehran University of Medical Sciences, Tehran 2193672411, Iran; d Department of Surgery, School of Medicine, Ahvaz Jundishapur University of Medical Sciences, Ahvaz 6193673111, Iran; e Persian Gulf Physiology Research Center, Medical Basic Sciences Research Institute, Ahvaz Jundishapur University of Medical Sciences, Ahvaz 6193673111, Iran; f Fertility, Infertility and Perinatology Research Center, Ahvaz Jundishapur University of Medical Sciences, Ahvaz 6193673111, Iran; g Translational Research Institute and Dermatology Institute, Academic Health System, Hamad Medical Corporation, Doha 3050, Qatar

**Keywords:** Leukemia, Leukemia stem cells, Pathogenesis, Signaling pathways, Stem cells

## Abstract

Leukemia is a malignancy in the blood that develops from the lymphatic system and bone marrow. Although various treatment options have been used for different types of leukemia, understanding the molecular pathways involved in the development and progression of leukemia is necessary. Recent studies showed that leukemia stem cells (LSCs) play essential roles in the pathogenesis of leukemia by targeting several signaling pathways, including Notch, Wnt, Hedgehog, and STAT3. LSCs are highly proliferative cells that stimulate tumor initiation, migration, EMT, and drug resistance. This review summarizes cellular pathways that stimulate and prevent LSCs' self-renewal, metastasis, and tumorigenesis.

## Introduction

Leukemia is a term for a group of lethal malignant diseases affecting blood and hematopoietic tissues (spleen, lymph nodes, and bone marrow).^1^ However literally, “leukemia” is a Greek word that means “white blood” and refers to the neoplastic proliferation of white blood cells or leukocytes.^2^ About 518,500 new cases of leukemia were globally diagnosed in 2017, showing that this disorder might become a significant concern for public health worldwide.^3^ Numerous factors such as chromosomal changes, genetic predisposition, radiation, chemical agents, immunodeficiency, and viruses are probably responsible for the pathologic feature of leukemia.^4^, ^5^, ^6^ Based on the clinical course of the disease and the cell of origin, there are four main types of leukemia, including acute myeloid leukemia (AML), acute lymphoblastic leukemia (ALL), chronic myeloid leukemia (CML), and chronic lymphocytic leukemia (CLL).^7^^,^^8^ In addition, there is a rare set of atypical leukemia with different clinical features.^9^ There are several treatment options for leukemia patients including radiation,^10^ chemotherapy,^11^ monoclonal antibodies,^12^ and transplantation of hematopoietic stem cells.^13^ Once the disease has occurred, several therapeutic procedures are determined based on the morphologic evaluation of blood cells and bone marrow specimens, analysis of cell-surface expression of cytoplasmic markers, identification of chromosomal abnormalities, or screening for molecular/cytogenetic markers.^14^ Therefore, understanding the molecular etiology and signaling pathways involved in the occurrence and progression of leukemia provides a potential avenue for future therapeutic research and treatment.^15^^,^^16^ Cancer stem cells (CSCs) are a small subgroup of tumor cells that play an essential role in tumorigenesis and tumor resistance.^17^ CSCs have self-renewal and differentiation properties.^18^ Indeed, the persistent abnormal self-renewal activity of CSCs leads to cancer development.^19^ CSCs are the main culprit for tumor metastasis to specific body areas and the heterogeneity of tumor cells.^20^^,^^21^

The self-renewal property of CSCs, disclosed by serial tumor transplantation refers to CSCs' ability to produce new stem cells via asymmetric or asymmetric divisions.^22^ Several signaling pathways are known as core actors of CSCs' self-renewal. For instance, the Wnt/β-catenin pathway is responsible for the proliferation, differentiation, maintenance, and regulation of cell stemness. Dysregulation of this pathway triggers the dedifferentiation of cancer cells and expression of specific CSC markers, leading to tumorigenesis.^23^ Besides, Hedgehog, PTEN, TGF-β, PI3K/Akt/mTOR, BMPs, and Notch 1-4/DLL/JAG are among other signaling pathways that play critical roles in the self-renewal, proliferation, and expression of CSC markers.^24^^,^^25^ Moreover, microRNAs (miRNAs) are regulatory elements of self-renewal activity and division of CSCs, which exert these functions through posttranscriptional gene silencing.^26^^,^^27^ For instance, the miR-34 family regulates CSCs' self-renewal by targeting the products of several genes including Notch, BCl2, E2F3, CDK-4, CDK-6, and HMGA-2.^28^

Leukemia stem cells (LSCs) have been identified in both acute and chronic myeloid types of leukemia.^29^^,^^30^ LSCs can hide from bone marrow treatments and resistance to conventional chemo and radiotherapy.^31^ Various genetic and epigenetic alterations, clonal diversification, and signaling molecules are involved in the activation of signal transduction in leukemia.^32^ Aberrant signal transduction augments the proliferation and survival of LSCs. Considerable investigations shed light on the necessity of identifying signaling molecules and pathways involved in LSCs' self-renewal, metastasis, and tumorigenesis.^33^^,^^34^ Targeting signaling molecules are potential therapeutic targets for developing targeted therapies for each type of leukemia.^35^^,^^36^ In this study, we reviewed the cellular pathways involved in the stimulation and prevention of LSCs' self-renewal, metastasis, and tumorigenesis.

## Cellular and molecular properties of LSCs

In 1994, LSCs were first identified in AML and then extended to a wide range of cancers. Dick and coworkers revealed that only the leukemic cells expressing CD34^+^/CD38^−^ markers, like normal adult hematopoietic stem cells, could induce hematopoietic malignancy and are called leukemia-initiating cells or LSCs.^37^ Recently, the origin of LSCs has gained much attention among researchers.^38^ It has been explained that random mutations in normal stem cells during DNA replication may convert these cells to CSCs.^39^ Moreover, some studies indicated that CSCs could be derived from mature cells via three critical mechanisms, including gene transfer, genomic instability, and microenvironment alteration.^40^, ^41^, ^42^ Cell fusion is another process that may trigger cancer initiation and progression.^43^^,^^44^ The fusion of tumor cells with lymphocytes may be responsible for tumor cells' genotypic and phenotypic diversity.^45^^,^^46^ More recently, several studies have declared that CSCs have special metabolic flexibility compared with cancer and normal cells.^47^^,^^48^ Thus, the metabolic reprogramming process may be another factor contributing to the development of CSCs.^49^ Whereas cancer and normal cells are glycolytic and undergo oxidative phosphorylation (OXPHOS), CSCs can shift between glycolysis and OXPHOS to keep homeostasis and induce tumor growth.^25^

More differentiated and mutated normal stem cells might generate a population that can be regarded as LSCs.^50^ Different characteristics of LSCs include self-renewal, high proliferation capacity, the ability to activate the NF-kappa B pathway, and the possibility to migrate when transplanted into a recipient mouse.^51^^,^^52^ LSCs express a set of cell surface markers that have the potential to be targeted for cancer therapy. According to the cell surface marker profile, LSCs are CD34^+^/CD38^−^/CD123^+^ malignant cells that initiate leukemia in NOD/SCID mice with unfractionated AML.^53^ CD34 is a cell surface glycoprotein associated with the therapy outcome and minimal residual disease level in LSCs.^54^^,^^55^ CD38 is introduced as a type II membrane glycoprotein correlated with the prognosis of LSCs.^56^ CD123 is the α-chain of the interleukin-3 receptor expressed in LSCs whose up-regulation can promote the departure of bone marrow-LSCs into circulation via down-regulating CXCR4.^57^^,^^58^

The CD33 antigen is highly expressed in AML blast that is limited to LSCs. This marker is an exciting target for AML therapy.^59^ CD33, a membrane-bound protein, belongs to the Siglec family and plays a critical role in the inflammatory response.^60^ LSCs in AML are phenotypically restricted to CD34^+^/CD38^**−**^ cells. Therefore, identifying CD34^+^/CD38^+^/CD19^+^ self-renewing B-ALL cells introduces a hierarchy of leukemia-initiating cells that differ from AML.^61^ Evidence showed that cells with a primitive CD34^+^/CD38^**−**^/CD33^**−**^/CD10^**−**^/CD19^**−**^ phenotype may cause B-cell precursor ALL and Philadelphia chromosome-positive (Ph^+^) ALL. The transformation process in the primitive hemopoietic cells may cause B-cell precursor ALL, CML, and AML. The transformed LSCs showed self-renewal capacity but revealed a limited differentiation ability and thus may be a critical factor in disease progression.^62^

**Figure 1:**
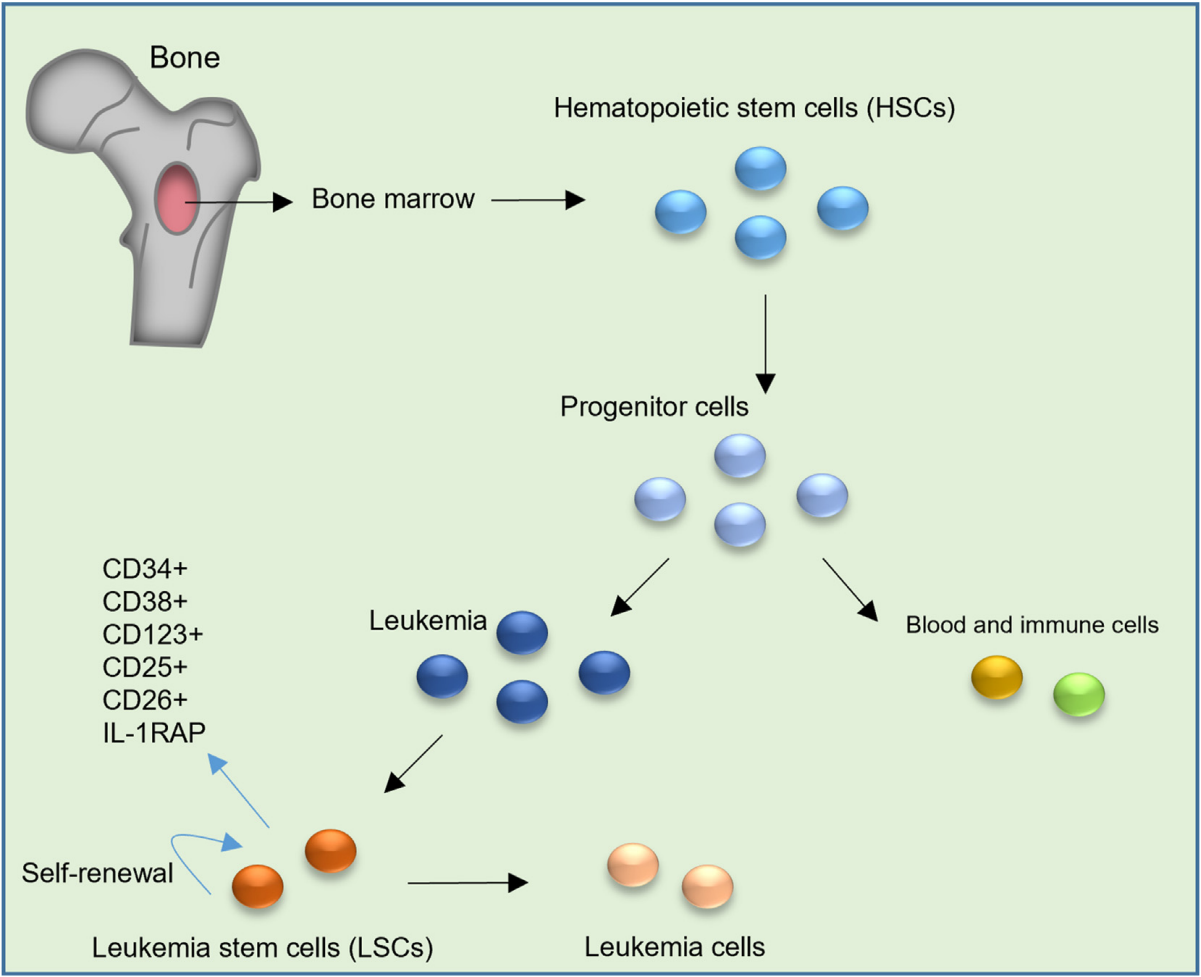
The origin and molecular properties of leukemia stem cells (LSCs).

CD25, CD26, and interleukin-1-receptor accessory protein (IL-1RAP) are other differentially expressed antigens of CML-LSCs.^63^^,^^64^ CD25 (IL2Rα) is modulated through STAT5 activity, and highly expressed CD25 is found to decrease the proliferation ability of CML-LSCs.^65^ Also, the binding of IL-1RAP as a co-receptor of IL-1 to CD25 facilitated CML-LSCs proliferation through activation of the NF-kβ and AKT pathways.^66^ Several findings proposed that the expression of CD25 and IL-1RAP are distinctive to CML-LSCs in the CD34^+^/CD38^−^ population.^67^^,^^68^ CD26 was shown as a multifunctional glycoprotein and a co-stimulator of T cell activation that cleaved the SDF1/CXCR4 axis to release bone marrow CML-LSCs into the blood.^50^^,^^69^ Further studies in terms of transcriptomics and proteomics combination may be an efficient approach to understanding expressed antigens in LSCs qualitatively or quantitatively.^70^ Figure 1 shows the origin and molecular properties of LSCs.

## DNA repair and epigenetic pathways involved in LSCs

To preserve genomic integrity, a complicated DNA repair system is developed to reciprocate different forms of DNA lesions, and these approaches are introduced as the DNA damage response (DDR).^71^ DDR deficiencies participate in cancer pathogenesis, and deficiencies in DDR signals provide curative chances to target malignant LSCs with minimum side effects on normal cells.^72^ Abnormal regulation of DNA repair elements can influence their stability, localization, activity, and interplay with DDR factors, which finally accelerates the chance of leukemia progression.^73^ Therefore, recognition of deregulated DNA repair molecules might be utilized in targeted curative approaches for leukemia.^74^ Inherited or acquired mutations or some polymorphisms in the DNA repair genes can make individuals susceptible to leukemia.^75^

Epigenetics is inheritable phenotype modifications beyond the DNA sequence.^76^ Those changes comprise DNA and histone modifications, known as epigenetic marks.^77^^,^^78^ Such markers are subjoined to particular DNA and histones through various enzymes.^79^ Modifications in the epigenomes participate in the instability of chromosomes, supply survival benefits to LSCs, and result in cancer initiation and progression.^80^ Although different studies have revealed that epigenetic modulators are required for normal development, the dysregulated DNA modification pattern is considered a chief characteristic of LSCs.^81^ Cytosine methylation in the CpG motifs is mediated by DNA methyltransferase enzymes called DNMTs.^82^ In addition to the role of DNMTs in hematopoiesis, they have also been implicated in hematological disorders, thereby being likely vital in LSCs.^83^ Accumulating evidence has connected DNMT deficiency to LSC expansion and further leukemogenesis.^84^ Noticeably, genomic profiling disclosed unpredicted widespread mutations in the DNA methylation factors like Ten-Eleven Translocation 2 (TET2).^85^ TET2 is the sole mutated gene that belongs to the TET family and is involved in various human hematological disorders.^86^ Interruption of TET2 is reported to expand multipotent and myeloid progenitors, resulting in the accumulation of pre-LSCs.^87^ Moreover, its disruption stimulated HSCs' self-renewal and generated a pre-LSC population.^88^^,^^89^ Besides, sustained TET2 deletion is necessary for the maintenance of pre-LSCs' self-renewal, while its impacts are inversed upon restoration of TET2.^90^ The greatest known histone modification includes the methylation of lysine (K) residues, which can be either switched on or inhibited, and the acetylation of K residues.^84^ Cancer genomics studies have revealed that more than 30% of AML patients show mutations on chromatin modifiers like Enhancer of Zeste Homolog 2 (EZH2).^91^ EZH2 can tri-methylate histone 3 at lysine 27 (H3K27) to induce transcriptional suppression of target genes.^92^ Its aberrant expression differentially participated in tumor initiation in various kinds of leukemia.^91^^,^^93^^,^^94^ Interestingly, high EZH2 expression is found in CML-LSC. It was shown that the survival of CML-LSCs depends on EZH2 expression and more precisely the enzymatic activity of EZH2, while its inactivation influences LSC survival and inhibits disease from initiation and maintenance.^95^ EZH2-regulated epigenetic under-expression of the insulin growth factor-1 (IGF1) pathway stimulated LSC activity.^96^ EZH2 can maintain LSCs by reinforcing their differentiation blockade by the modulation of its direct targets.^85^ A major deal of effort is ongoing to discover drugs capable of reversing particular histone methylation marks.^97^ A novel class of oligoamine analogs was identified as potent inhibitors of lysine-specific demethylase 1 (LSD1).^98^ The capability of LSD1 to influence both DNA and histone methylation suggests it is a novel target for epigenetic treatment.^99^

It has been found that miRNAs as small non-coding RNAs (ncRNAs) (19−25 nt) enable another aspect of epigenetic control of leukemogenesis.^100^ MiRNAs' cooperation with DNA methylation could modulate the balance between self-renewal and differentiation of LSCs.^84^ For instance, miR-130 b and miR-181 up-regulation in LSCs increased self-renewal and tumorigenicity.^101^^,^^102^ Epigenetic modifications are early incidents within LSC formation but epigenetic genes have been regarded as attractive targets for novel treatments due to the reversible modality of epigenetic signatures.^85^^,^^103^ For instance, targeting EZH2 using a particular shRNA showed a significant inhibitory impact on LSCs and extremely extended the survival of CML animal models.^94^ Besides, other ncRNAs, including circular RNAs (circRNAs)^104^ and long non-coding RNAs (lncRNAs),^105^^,^^106^ by targeting several genetic and epigenetic factors, are involved in the stimulation or suppression of leukemia progression, thereby providing a possible curative approach for leukemia. In this review, we focused on the potential roles of miRNAs in the stimulation or suppression of LSCs.

## Critical signaling pathways involved in LSCs

By targeting several signaling pathways, including Notch, Wnt, PI3K, Hedgehog, TGFβ, and STAT3, LSCs play essential roles in the pathogenesis of leukemia^107^^,^^108^ (Table 1). Here, we summarize multiple pathways essential for LSCs.

**Table 1:** Functional roles of leukemia stem cells (LSCs) in leukemia.

Pathway	Maintenance/stemness	Apoptosis	Chemosensibility/resistance	Development/metastasis	Self-renewal	Differentiation/proliferation	Reference
Notch	yes	yes	yes	yes	–	yes	^109^,^112^,^113^,^220^,^256^
Hedgehog	yes	yes	yes	yes	–	yes	^119^,^120^,^122^,^123^,^128^
Wnt/β-catenin	yes	–	yes	yes	yes	yes	129, 130, 131, 132^,^^139^,^140^,^257^
JAK/STAT	yes	yes	–	–	yes	yes	^181^,^183^,^185^
KDM4C	yes	–	–	yes	–	yes	^178^
FoxO	–	–	–	–	yes	–	^117^
ERK-MSK MAPK	–	yes	yes	yes	–	–	^214^
NF-κB	–	yes	yes	–	–	–	^197^
BMI-1	yes	yes	–	–	yes	yes	^143^,^146^
SIRT1/TSC2	–	–	–	yes	–	yes	^201^,^204^
TGFβ	–	–	yes	–	–	yes	^148^,^151^
Interleukin	yes	–	–	yes	–	yes	188, 189, 190
Alox5	–	–	–	yes	–	–	^219^,^220^
PDK1	yes	yes	–	–	–	–	^148^
PTEN	yes	–	–	yes	–	–	^158^
NF-κB	–	yes	yes	–	–	–	^197^
IGF2/IGF1R/Nanog	–	yes	–	yes	–	yes	^207^,^209^
Gas6/AXL	–	–	–	yes	yes	–	^171^
AHR	yes	–	–	–	yes	yes	^174^
LIGHT/LTβR	–	–	–	–	yes	–	^167^
TNF-α	yes	–	–	–	yes	–	^194^
FcγRIIb	–	–	yes	–	–	yes	^211^
PLCG1	yes	–	–	–	yes	yes	^168^
Myc-Miz1	–	–	–	yes	yes	yes	^163^
Sphingolipid	–	–	–	–	–	yes	^198^

## Signaling pathways governing LSC maintenance

### Notch pathway

Dysregulation of the Notch signaling pathway was reported to transform adult HSC into a preleukemic state.^109^ Notch 1 mutation has been reported in more than 50% of T-ALL patients but its prognosis remains unknown and appears to be associated with additional genetic lesions.^110^^,^^111^ In hematological neoplasia, Notch 3-induced JAG1 as a paracrine stimulator facilitated leukemic cell survival, proliferative, and invasive ability and participated in the pathogenesis of T-ALL.^112^^,^^113^ In T-cell progenitors, the Notch pathway induced LSC activity.^114^ Notch activation could stimulate differentiation, apoptosis, and cell-cycle arrest in AML-LSCs.^115^ This pathway in a regulatory loop with the Wnt pathway influenced LSC maintenance in T-cell ALL. The Notch family components including Notch 1 and Notch 2 are considered the top possible targets for controlling the proliferation and metastasis of T-ALL-LSC.^21^ Therefore, several single-cell technologies, conventional chemotherapy, and targeted treatments toward Notch suppression could reduce LSC quiescence, facilitate LSC chemosensitivity, and eradicate LSCs.^113^ Epigenetic recovery of the Notch1-driven autocrine IGF1 pathway was also implicated in the repressed activity of LSCs.^96^ Moreover, targeting the Musashi2-Numb pathway through the main genes of the Hedgehog and Notch pathways is an efficient curative approach for chronic LSCs.^116^ Although the correlation of the Notch pathway in AML-LSCs' self-renewal has not been definitely exhibited, Notch in cross-talk with the Wnt pathway had a positive effect on Wnt-dependent AML.^117^

### Hedgehog pathway

Abnormal activation of the Hedgehog pathway has been reported in LSCs but Hedgehog was found unessential for maintaining AML-LSCs.^109^^,^^118^ In contrast, another study demonstrated that the Hedgehog pathway and the Hedgehog components, including Smoothened (Smo) and GLI1 are involved in the stemness and survival of AML-LSCs.^119^ Therefore, this pathway seems to be implicated in the progression of drug resistance.^120^ Smo, as a key transmembrane protein, can sensitize AML-LSCs to chemotherapy and accelerate drug resistance.^121^ The combination of chemotherapy with Hedgehog signaling antagonists decreased the dormancy of LSCs and facilitated their differentiation.^119^ This signaling controls LSC frequency and the maintenance of blast crisis (BC).^122^^,^^123^ LSC persistence is responsible for relapse in AML, and blockade of Hedgehog forced LSC entry into the cell cycle, leading to greater chemosensitivity.^124^^,^^125^ Glasdegib is a Smo inhibitor that targets the Hedgehog function in LSCs.^125^ Smo knockdown has been implicated in decreasing CML-LSCs' pathogenesis.^123^ LDE225, a Smo antagonist, combined with nilotinib, a tyrosine kinase inhibitor, may introduce a novel approach to induce the eradication of CML-LSCs.^126^^,^^127^ Moreover, mesoporous silica nanoparticles carrying siRNA_GLI1_ and siRNA_SMO_ have been shown to facilitate LSCs' apoptosis and may be regarded as a chemotherapeutic drug cocktail to manage leukemia.^128^

### Wnt/β-catenin pathway

The Wnt/β-catenin signaling plays an essential role in LSC development.^129^ Activating β-catenin as a member of the Wnt/β-catenin signaling is known to improve MLL-LSCs. Blockade of β-catenin inversed LSCs to the pre-LSC-like stage and dramatically decreased the properties of LSCs, including cell growth, self-renewal, leukemia formation, and response to GSK9 antagonist therapy. Therefore, understanding β-catenin function in MLL-LSCs highlighted this pathway as a possible therapeutic target for the selective elimination of AML-LSCs.^130^, ^131^, ^132^ Suppression of COX as an abrogator of β-catenin in fully developed MLL-AF9-induced leukemia decreased both β-catenin and the frequency of LSCs. It can be concluded that some subtypes of AML-LSCs are dependent on the Wnt pathway, participating in the self-renewal ability of LSCs.^109^^,^^133^ WNT974 is an inhibitor for the palmitoylation of Wnt ligands and a target for CML-LSCs.^134^ WNT974-treated leukemia cells decreased Wnt targets, Wnt signaling activity, and *in vitro* self-renewal for primary AML-LSCs. *In vivo* studies showed that WNT974 therapy may not affect LSCs' function. Besides, WNT974, combined with other active agents in AML microenvironment, could maintain Wnt targeting and consequent elimination of AML-LSCs.^135^^,^^136^ Telomerase complex has been implicated in the self-renewal of the Wnt/β-catenin pathway. In the LSC microenvironment, telomerase complex combined with the Wnt/β-catenin pathway sensitizes β-catenin-activated LSCs to imetelstat as a competitive antagonist of telomerase activity in the *in vitro* and *in vivo* CML models, providing an intriguing approach for LSC elimination.^137^ In contrast, in another recent AML mouse model study, β-catenin did not affect the self-renewal ability of AML-LSCs. Therefore, β-catenin targeting may not be as efficient as previously reported for eliminating AML-LSCs.^138^ Therefore, the dual function of the Wnt/β-catenin pathway for eradicating LSCs needs more investigation. Moreover, high expression of RSPO-LGR4 as a positive regulator of the canonical Wnt/β-catenin pathway is required for AML-LSCs' self-renewal. RSPO-LGR4 blockade abolished the leukemia-initiating capacity of LSCs by inducing their differentiation without damaging the normal stem cell compartment, thereby highlighting a therapeutic window to definitely target LSCs.^139^ GPR84 is a member of the *G* protein-coupled receptor family and a new β-catenin modulator that is defined to maintain fully progressed AML via retaining dysregulated β-catenin in LSCs.^140^ Besides, targeting CD marker signaling, including CD27 on LSCs, may demonstrate an intriguing therapeutic insight into inhibiting the Wnt/β-catenin pathway in CML.^141^ In addition, other pathways such as SDF1/CXCL12, VCAM/VLA-4/NF-κB, CD44, and hypoxia play essential roles in drug resistance.^142^

### BMI-1 pathway

Oncogene BMI-1 is a polycomb group (PcG) RING-finger protein required for LSCs' maintenance and self-renewal.^143^ BMI-1-deficient mice showed engraftment and proliferative disability and stimulated differentiation and apoptosis in AML stem/progenitor cells.^144^^,^^145^ BMI-1 and Ring1b can organize a heterodimeric complex that is involved in the initiation and maintenance of LSCs. Epigenetic inhibition of BMI-1, including methylation, histone deacetylase, and ubiquitin-proteasome could be utilized as anti-BMI-1 insights in LSCs.^146^

### TGF-β pathway

TGF-β has a dual role in cancer cells.^147^ Several reports implicated that TGF-β had a protective role in myeloid LSCs through modulating downstream parallel signaling required for cell proliferation.^148^ Other studies suggested that TGF-β can function as an oncogene in LSCs. TGF-β is a pivotal modulator of AKT activity, and AKT-dependent repression of FOXO3a is necessary for CML-LSCs' elimination.^149^ In AML, the hypoxic niche of bone marrow is reinforced by leukemic cells and stimulated TGF-β activity.^150^ TGF-β can elevate CXCR4 expression and facilitate the resident chemo-resistant survival of LSCs.^151^ Dysregulation of the PI3K/Akt/mTOR pathway (phosphoinositide 3-kinase (PI3K)/Akt/mammalian target of rapamycin (mTOR)) as one of the TGF-β intracellular signals has been reported in leukemia.^152^ Members of the forkhead O (FoxO) transcription factor is an effector of the PI3K-AKT signaling that is dysregulated in hematologic disorders.^153^ In FoxO3a-deficient mice, serial transplantation of leukemia-initiating cells reduced the LSC population in the CML mouse model which was more notable by imatinib as a tyrosine kinase inhibitor.^149^ It has been found that FoxO3a deficiency influences LSCs' self-renewal.^117^ Moreover, the *in vivo* AML model has revealed that FoxO knockdown could selectively reduce LSCs, providing a promising molecular marker in AML management.^154^ FoxOs are the primary modulators of reactive oxygen species (ROS) homeostasis.^155^ It has been suggested that LSCs' self-renewal may be influenced by increased ROS levels.^156^ Therefore, targeting FoxO by affecting the upstream PI3K-AKT pathway could be a therapeutic mechanism to decrease the LSC burden.^117^^,^^157^ Phosphatase and tensin homolog (PTEN) is a direct target of PI3K that regulates cell survival by activating pyruvate dehydrogenase kinase 1 (PDK1).^158^ Deletion or inactivation of PTEN is reported in hematological disorders.^159^ The activation of PTEN repressed CML-LSCs and promoted cell cycle arrest. In contrast, low expression of PTEN facilitated CML progression, while PTEN overexpression postponed disease progression by inhibiting LSC function. Therefore, PTEN has a tumor suppressor role in myeloid LSCs.^158^ It has been demonstrated that PDK1 loss of function increased survival due to elevated LSC apoptosis in the AML murine model. Hence, PDK1 presented a crucial role in LSC maintenance.^148^ The PI3K/AKT/mTOR pathway is the upstream regulator of c-Myc.^160^ Aberrant expression of c-Myc is required for tumor cell proliferation and survival.^161^ Max-regulated transactivational and Myc-interacting zinc finger protein 1 (Miz1) can play an important role in the activation of Myc. AML cells expressing MycV394D (intrinsic Myc deleted) are partly differentiated and decrease the colony-forming ability *in vitro* and the leukemogenic capacity *in vivo*. Low levels of LSCs in MycV394D-AML cells proposed that Myc-Miz1 binding is necessary for LSCs' self-renewal.^162^ Myc suppressed Miz1-regulated CCAAT/enhancer-binding protein α (Cebpα) and its expression.^162^ Therefore, this pathway has a critical role in AML progression by targeting LSCs' self-renewal along with their undifferentiated state.^163^ Hypoxia-inducible factor 1α (HIF-1α) is highly expressed in LSCs and is necessary for AML-LSCs' survival and maintenance.^164^ Its suppression induced LSCs' apoptosis.^164^ In T-ALL, HIF-1α accelerated the frequency of LICs by targeting β-catenin^108^. HIF-1α in combination with the Wnt/β-catenin pathway supports the T-ALL-LSC function^165^ and can be regulated with the PI3K/AKT pathway.^166^

### LIGHT/LTβR pathway

Lymphotoxin-β receptor (LTβR) is a tumor necrosis factor-alpha (TNFα) receptor superfamily member and its loss of function has been shown to decrease LSCs numbers and extend survival in a CML animal model. The LTβR pathway in combination with LIGHT induced the colony-forming capacity in human G-CSF mobilized HSCs and human LSCs.^167^ The LIGHT/LTβR pathway has been reported to modulate LSCs' and HSCs' quiescence and self-renewal through decreasing cell proliferation and symmetric cell division over asymmetric ones. Since asymmetric division resulted in stem cell differentiation, LIGHT/LTβR targeting may suggest a promising insight to facilitate differentiation and eradicate LSCs.^108^^,^^167^

### PLCG1 pathway

In AML1-ETO (AE)-driven AML, phospholipase C gamma 1 (PLCG1) is an AE fusion protein-specific target that is increased after binding of AE to intergenic modulatory DNA elements.^168^ Genetic inactivated PLCG1 in animal and human AML has been demonstrated to repress *in vivo* self-renewal and leukemic proliferation and maintenance in AML1-ETO. In contrast, PLCG1 was inessential for the function of normal hematopoietic and progenitor stem cells. Such evidence indicates that the PLCG1 signaling serves as a main curative target for AML1-ETO LSCs.^168^

### Gas6/AXL pathway

As a member of the TAM receptor tyrosine kinases (TAMR), AXL and its ligand, growth-arrest-specific gene 6 (Gas6), were found to participate in LSC pathogenesis.^169^^,^^170^ High expression of AXL was reported in primary CML CD34^+^ cells, and its blockade decreased CML-LSCs' survival, self-renewal, and maintenance. Recruitment of bone marrow-derived stromal cells (BMDSCs) and primary mesenchymal stem cells (MSCs) with human CML CD34^+^ cells led to secreting Gas6 to induce LSCs' self-renewal. By interaction with AXL, Gas6 stabilizes β-catenin in an AKT-dependent manner in human CML-LSCs. Therefore, Gas6/AXL is a therapeutic axis for CML-LSCs' elimination.^171^

### AHR pathway

Low expression of the Aryl hydrocarbon receptor (AHR) pathway has been demonstrated in human AML and LSC-enriched populations.^172^^,^^173^ AHR is a tumor suppressor and an inducer of differentiation which is suppressed in human AML blasts and more favorably underexpressed in LSC-enriched populations within leukemias. In particular, AHR agonist FICZ has been informed to destroy leukemic growth, induce differentiative potential, and suppress self-renewal. FICZ induction has no adverse effect on normal hematopoietic stem and progenitor cells (HSPCs) and could not overexpress an eminent LSC-particular AHR target in HSPCs.^174^

### PDK1 pathway

It has been demonstrated that Pyruvate dehydrogenase kinase 1 (PDK1) loss of function increased survival due to elevated LSCs' apoptosis in the AML murine model.^175^ Hence, PDK1 presented a crucial role in LSCs' maintenance.^148^

### KDM4C/ALKBH5/AXL pathway

N6-methyladenosine (m6A) is a kind of common modification of mammalian mRNAs that affects various cellular processes, and such modification is catalyzed by AlkB Homolog 5 (ALKBH5) demethylase.^176^ ALKBH5 is modulated by lysine demethylase 4C (KDM4C) as a histone demethylase.^177^ AXL is a member of the receptor tyrosine kinase (RTK) family, and its constitutive activation has been reported in AML.^177^ It has been shown that expression of m6A demethylase ALKBH5 is modulated through chromatin state changes within human AML leukemogenesis and ALKBH5 is indispensable for LSC maintenance but it is not necessary for normal hematopoiesis. KDM4C modulated the expression of ALKBH5 by promoting chromatin accessibility of ALKBH5 locus, decreasing the level of H3K9me3, and inducing MYB and Pol II recruitment. The relation between chromatin state dynamics and expression modulation of m6A modifiers highlighted the pivotal function of ALKBH5 in AML.^178^

### Inflammatory pathways involved in LSCs

#### JAK/STAT pathway

High expression of STAT3 is associated with shorter survival and poor clinical outcome in BMDSC, which is correlated with disease-initiating stem cells.^179^ Inhibition of STAT3 by AZD9150, an antisense STAT3 inhibitor, decreased viability along with *in vivo* leukemic growth and induced LSCs' apoptosis.^180^ Incorporation of AZD9150 with AML/MDS stem cells induced hematopoietic differentiation. As a result, antisense oligonucleotide-regulated aberrant activation of the STAT3 pathway can be a promising approach to diminish AML and MDS stem cells.^181^ Moreover, STAT5 has been shown to be involved in LSCs' self-renewal.^182^ Hence, managing aggressive leukemia needs the repression of several pathways.^183^ The Janus kinase (JAK) pathway in combination with STAT signals, is overexpressed in LSCs.^184^ The JAK2 blockade was found to decrease AML-LSCs' growth. The JAK/STAT pathway has an essential role in AML-LSCs by targeting various growth factor receptors.^185^ Bone morphogenetic protein receptor type-1B (BMPR1B) as a stem cell modulator also affect persisting and dormant LSCs invisible in their BM niche.^186^

#### Interleukin pathway

Tregs are one of the leading causes of immunosuppression in the bone marrow niche.^187^ Evidence has demonstrated that interleukin 10 (IL-10) as an anti-inflammatory cytokine is released by Tregs-induced AML-LSC stemness through activating the PI3K-AKT pathway. The IL-10 interaction with its receptor, IL10R or PI3K-AKT blockade, has been reported to decrease AML-LSC stemness. Therefore, targeting Tregs/LSC may inhibit AML progression.^188^^,^^189^ Moreover, the IL-1 receptor antagonist can interact with tyrosine kinase inhibitors in CML-LSC elimination. It has been proved that IL-1β facilitates LSCs' proliferation and maintenance. However, the function of the IL-1 pathway in pre-LSC emergence along with AML progression is less clear.^190^ Previous studies have found IL-6 functions in pre-LSCs and LSCs.^191^ In addition, high expression of IL-8 along with CXCR2 as its receptor has been reported in LSCs from MDS patients.^192^ Such interleukins could be regarded as an intriguing biomarker in LSC therapy.

#### TNF-α pathway

The bone marrow mesenchymal microenvironment has an essential role in LSC maintenance.^193^ High expression of CXCL1 and its receptor CXCR2 were shown in LSCs.^194^ CXCL1 facilitated LSC proliferation along with its self-renewal. CXCR2 blockade decreased LSC growth and induced LSC targeting by interaction with tyrosine kinase inhibitors (TKIs).^195^ Therefore, changes in TNF-α in the BM-CML stromal microenvironment promoted LSC maintenance and growth with the CXCL1-CXCR2 pathway, and CXCR2 suppression efficiently inhibited CML-LSCs.^194^

#### NF-κB pathway

Primitive AML cells showed aberrant expression of NF-κB; thereby, targeting this factor may provide novel insights to ablate LSCs preferentially.^196^ Dysregulated activation of the NF-κB pathway has been shown in LSCs' drug resistance; NF-κB inhibition efficiently induced LSCs' drug resistance and increased K562/ADM cell sensitivity to doxorubicin-induced apoptosis.^197^ The NF-κB pathway blockade along with low expression of programmed cell death ligand 1 (PD-L1) via interleukin-1 receptor-associated kinase 1/4 (IRAK1/4) and imatinib, has been reported to repress CML-LSCs. Collectively, IRAK1/4 inhibitors via TKIs are an intriguing insight into achieving CML-LSC therapy.^29^ Sphingosine-1-phosphate receptor 3 (S1PR3) is a leading downstream pathway in the TNFα–NF-κB axis that modulates AML-LSC differentiation and activates the inflammatory programs.^198^ Hence, regulating the sphingolipid pathway by S1PR3 may improve clinical outcomes in patients with leukemia.^198^

#### SIRPα pathway

The interaction of signal-regulatory protein alpha (SIRPα) and CD47 as its ligand was determined through Ig variable region (IgV)-like domains.^199^ Following CD47 binding, the SIRPα immunoreceptor tyrosine-based suppression motifs regulated some inhibitory pathways. Experiments on animal model expressing SIRPα variants with the differential binding capacity to human CD47 exhibited that macrophage-regulated phagocytosis and AML-LSC clearance was hinged on the absence of the SIRPα pathway. In the AML-xenotransplant model, LSCs' function depended on SIRPα-regulated suppression in macrophages by CD47 engagement. Moreover, SIRPα-Fc therapy has been shown to induce AML cell phagocytosis via mouse and human macrophages and destroyed mice leukemic engraftment. SIRPα-Fc therapy could not elevate the phagocytosis of normal hematopoietic targets. Thereby, inhibition of the SIRPα pathway increased macrophage-regulated eradication of AML-LSCs.^200^

### Signaling pathways involved in LSCs proliferation

#### SIRT1/TSC2 pathway

Since the discovery of LSCs' involvement in AML relapse and refractory, ongoing research focused on natural products targeting multiple signaling pathways modulating the pathogenesis of LSCs.^201^ Ginsenoside Rg1 (Rg1) is an active component in ginseng that suppresses radiation resistance.^202^^,^^203^ A recent study revealed that Rg1 repressed the proliferative ability and facilitated cell cycle arrest in CD34^+^/CD38^−^/AML^−^ LSCs through a significant increase of CD34^+^/CD38^−^ LSCs derived from human AML cells in the G0/G1 phase and their dramatic reduction in the G2/M and S phases.^204^ Sirtuin 1 (SIRT1) and tuberous sclerosis complex 2 (TSC2) participated in cell senescence.^205^^,^^206^ Moreover, Rg1 significantly increased mixed colony-forming unit and senescence-associated beta-galactosidase as cell senescence markers along with a significant reduction in SIRT1 and TSC2 expression in CD34^+^/CD38^−^ LSCs derived from human AML cells.^204^ Rg1 remarkably induced cell senescence indicators of CD34^+^/CD38^−^ AML-LSC by activating the SIRT1/TSC2 pathway.^204^ Future research should be handled to survey the impacts of Rg1 on LSCs both *in vitro* and *in* vivo.

#### IGF2/IGF1R/Nanog pathway

Silencing of Nanog as a transcription factor for LSCs has been demonstrated to suppress the proliferative ability and facilitate apoptotic and cell cycle arrest potential of AML-LSC which is modulated with the insulin-like growth factor receptor (IGF1R) pathway.^207^ The binding of IGF1R to IGF1 and IGF2 activated IGF1R activity.^208^ By contrast, Nanog silencing terminated IGF2 effects on the colony formation ability of AML-LSCs. Some studies proposed that the IGF2/IGF1R/Nanog pathway presented a pivotal function in LSC proliferation.^207^ Recently, functional assays exhibited that high miR-150 expression suppressed the proliferative potential and clonogenic growth, increased chemosensitivity, and ameliorated *in vitro* tumorigenicity of CD34^+^/CD38^−^ AML-LSCs. *In vivo* animal model experiments also showed that miR-150 up-regulation progressively canceled tumor growth. Moreover, Nanog's loss of function repeated the anti-proliferation and tumorigenicity suppression impacts. In addition, miR-150 directly underexpressed other cancer stem cell markers such as Notch 2 and CTNNB1. Nanog has been considered a direct and functional target of miR-150 and its particular biological behavior in modulating the proliferative capacity and tumorigenicity of LSCs highlighted its importance in AML-LSCs.^209^

#### FcγRIIb pathway

Despite the advancement in targeted molecular suppression of the oncogenic driver BCR-ABL in CML, the greater numbers of patients still need to prolong the treatment of tyrosine kinase inhibitor (TKI).^210^ Immunoreceptor tyrosine-based inhibition motif (ITIM) containing Fc gamma receptor IIb (FcγRIIb, CD32b) has been regarded as a pivotal factor in LSC resistance. Targeting the FcγRIIb downstream pathway is a helpful therapeutic strategy.^211^ Overexpression of FcγRIIb was identified in primary CML-LSCs, and its loss of function decreased serial re-plaiting efficiency and cell proliferative ability in CML-LSCs. Transgenic and retroviral CML animal model experiments highlighted that FcγRIIb targeting successfully reduced *in vivo* CML-LSCs. BTK is a principal downstream regulator that targets the BCR-ABL-FcγRIIb-BTK axis in primary CD34^+^ CML cells. The combination of BTK with standard TKI treatment has been reported to induce apoptosis in quiescent CML-LSCs. Therefore, combining BCR-ABL-TKI therapy and BTK repression may be a suitable strategy against LSCs.^211^

### Other signaling pathways in LSCs

#### ERK/MSK/MAPK pathway

Survivin is one of the inhibitors of the apoptosis protein family that plays an essential role in numerous disorders.^212^ Survivin has correlated with worse prognosis, drug resistance, and poor overall survival.^213^ Survivin was overexpressed in LSCs and induced anti-apoptotic signals and resistance to chemotherapy.^214^ Downstream targets of the mitogen-activated protein kinase (MAPK) signaling, including ERK, JNK, and P38 have been implicated in tumor development.^215^^,^^216^ Highly-expressed survivin has been reported in CD34^+^/CD38^−^ AML-LSCs and paired CD34^+^ AML patients. Functional assay implicated survivin in LSCs drug resistance, and Sp1 and c-Myc simultaneously modulate survivin transcription levels. Clinically, Sp1 and c-Myc have been found to be overexpressed and showed a positive correlation with survivin in CD34^+^ AML patients. Moreover, the ERK/MSK signaling activated Sp1 and c-Myc expression.^214^ MSK is a downstream target of ERK that was modulated by the MAPK/ERK signal.^217^ Therefore, ERK/MSK/Sp1/c-Myc network served as a pivotal modulator of survivin expression in LSCs, suggesting a possible novel curative strategy for LSC management.^214^

#### Alox5 pathway

The Arachidonate 5-lipoxygenase (5-LO) (Alox5) gene is a pivotal modulator of CML-LSCs. Without Alox5, BCR-ABL impairs promoting CML due to LSC function's impairment. Imatinib has been shown to efficiently repress BCR-ABL kinase activity without any effect on its protein which may partly inform why imatinib does not eliminate CML-LSCs. The total number and percentage of bone marrow CML-LSCs in mice were reported to be gently elevated with time during imatinib therapy.^218^ Alox5 signaling has been primarily correlated to the activation of β-catenin. Alox5 deletion underexpressed β-catenin in CML-LSCs but not in normal HSCs.^219^ The significance of Alox5 in modulating LSC function in CML is considered a promising therapeutic target in CML-LSCs.^219^^,^^220^

#### PTEN pathway

Deletion or inactivation of the phosphatase and tensin homolog (PTEN) is reported in multiple cancers such as hematological disorders.^159^ The activation of PTEN repressed CML-LSCs and promoted cell cycle arrest. In contrast, PTEN is underexpressed by BCR-ABL which facilitated CML progression, while overexpressed PTEN postponed disease progression by inhibiting LSC function. High PTEN expression postponed B-ALL development by Akt 1 as its main downstream target. In addition, the suppression of mTOR by rapamycin repressed human CML proliferation and CML-LSCs in mice. Such evidence supports the significance of the PTEN/PI3K/AKT/mTOR signaling in curing B-ALL, which is resistant to imatinib treatment. Therefore, PTEN has a tumor suppressor role in myeloid LSCs.^158^

### Potential roles of ncRNAs in LSCs

Previous studies revealed that ncRNAs such as miRNAs, lncRNAs, and cirRNAs play essential roles in stimulating or suppressing pathogenesis in LSCs.^221^, ^222^, ^223^, ^224^ It has been demonstrated that various lncRNAs, including HOXA10-AS, DANCR, HOTAIR, LAMP5-AS1, Morrbid, LINC00152, HOTTIP, MAGI2-AS3, and KIAA0125 participated in the survival, proliferation, and differentiation of LSCs. Besides, some studies have explored the effects of multiple cirRNAs such as circ_0040,823, circ_0004277, circCRKL, circ_0005774, and Hsa-circ_0003420 in LSCs' properties.^225^, ^226^, ^227^, ^228^ Here, we summarized the emerging roles of some miRNAs that regulate the progression of LSCs.

### miRNAs governing stimulation or suppression of tumorigenesis in LSCs

**Figure 2:**
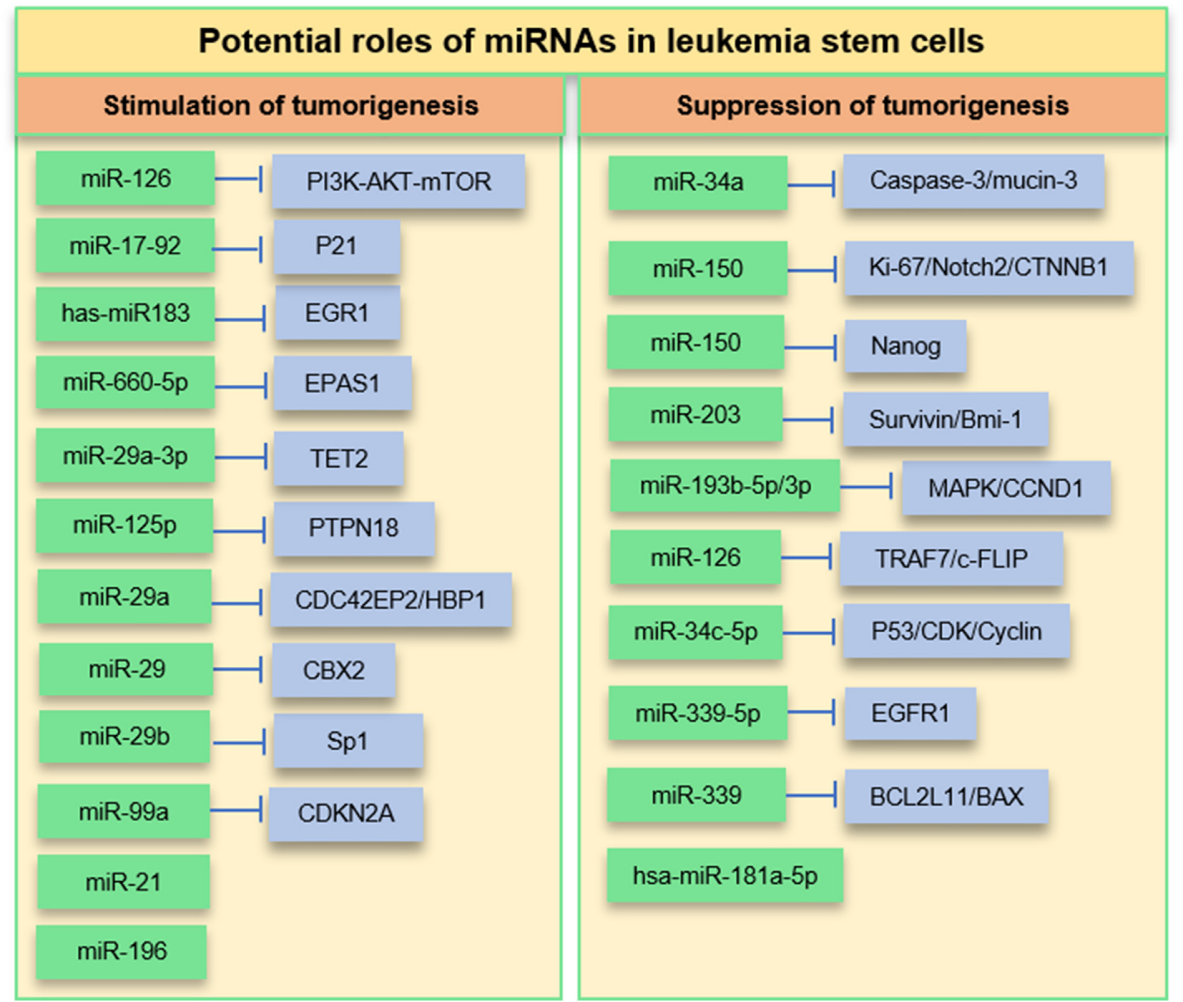
microRNAs play pivotal roles in stimulating or suppressing leukemia stem cell (LSC) pathogenesis.

Accumulating evidence shows that through several mechanisms, miRNAs have pivotal roles in the stimulation or suppression of LSCs.^229^ By targeting various biological processes, miRNAs are key regulatory molecules in cancer cells.^230^ Figure 2 lists several miRNAs involved in stimulating or suppressing LSC tumorigenesis.

### Stimulation of tumorigenesis

Several studies showed that miRNAs display regulatory roles in LSCs' self-renewal, metastasis, and drug resistance. High expression of miR-126 has been correlated with enhanced LSCs, poor survival, and high relapse AML.^231^ It has been found that by inhibiting the PI3K-AKT-mTOR pathway, miR-126 contributes to the G0-G1 cycle control and promotes the quiescence, self-renewal, and chemotherapy resistance of LSCs. Nanoparticles containing antagomiR-126 as inhibitors of miRNA-126 resulted in a depletion quiescent cell subpopulation of LSCs.^232^ Also, miRisten, as a novel inhibitor of miR-126 decreased leukemia burden and LSC activity in AML xenograft models.^233^ miR-17-92 overexpression in LSCs by reducing the expression of p21 regulated the arrested differentiation and increased the cellular proliferation of MLL-LSC.^234^ High expression of has-miR 183 through the BCR-ABL1 pathway in PH^+^ cells, by inhibiting early growth response 1 (EGR1) and consequently via enhancing the cell cycle regulator E2F1, directly regulated both cell proliferation and p53-dependent/independent apoptosis.^235^ Therefore, this miRNA could play a pivotal role in the proliferation and survival of CML-LSCs.^236^ Another investigation showed that high expression of miR-660–5p by targeting EPAS1 conferred TKI resistance to CML-LSCs *in vitro*.^237^ Some findings revealed overexpression of miR-29a-3p in CML-LSCs by binding to the 3′UTR region of TET2 (tet methylcytosine dioxygenase 2) and antioxidant-coding EPAS1 induced the protection of cells from imatinib mesylate (IM)-induced apoptosis.^238^ It has been shown that high miR-125 b could increase the tyrosine phosphorylation of GSK3 by inhibiting PTPN18, which is known as tyrosine phosphatase that dephosphorylates auto-phosphorylated kinases such as Her 2 and Abl, enhancing LSC frequency and self-renewal.^239^ Also, by targeting the cell cycle progression-related genes such as CDC42EP2 and HBP1, miR-29a accelerated the G1 to S/G2 cell cycle transitions and the self-renewing properties of AML-LSCs.^240^ Another study demonstrated that by targeting CBX2, miR-29 promoted the activity of AML-LSCs.^241^ miR-29 b was found to target specificity protein 1 (Sp1) to induce fucosyltransferases 4 (FUT4) transcription and stimulate the malignant behaviors of AML-LSCs.^241^ The miR-29 b/Sp1/FUT4 axis has promotional effects on AML-LSCs' progression through fucosylated CD44-mediated Wnt/β-catenin signaling.^242^ The suppression of miR-196 and miR-21, which are transcriptional targets of HOX-based leukemia oncoproteins could decrease human MLL-LSCs in the experimental model.^243^^,^^244^ Overexpression of miR-99a mediated stabilizing or activating p53 through up-regulation of CCNE1 and down-regulation of CDKN2A, which was correlated with LSC activity and worse survival in AML patients.^245^

### Suppression of tumorigenesis

Some investigations revealed that dysregulation of miRNA contributed to cancer stem cell proliferation and tumorigenicity.^246^ Some miRNAs are LSC suppressors or powerful tools in combat with hematological malignancies.^234^ Via releasing microvesicles, LSCs can promote the survival and migration of AML cells and regulate AML malignancy.^247^ It has been found that overexpression of miR-34a can inhibit this effect of LSCs by altering the downstream target genes, including caspase-3 and T-cell immunoglobulin mucin-3. miR-34a is a tumor-suppressive molecule associated with AML cell growth and invasion.^248^ miR-150 is another molecule down-regulated in LSCs.^249^ Overexpression of miR-150 attenuated LSCs' proliferation, clonogenicity, and tumorigenicity and enhanced the chemosensitivity of tumors. miR-150 through overexpression of caspase-3 and down expression of Ki-67, Notch 2, and CTNNB1 regulated LSCs' proliferation and tumorigenicity.^249^ Moreover, through targeting the Nanog protein, miR-150 could exert inhibitory effects on the proliferation and tumorigenicity of LSCs. Nanog is another known target of miR-150 that is overexpressed in cancer stem cells and enhances the proliferation, invasion, and resistance of tumor cells.^209^ It has been reported that miR-203 is down-regulated in multiple cancers including AML, which contributes to the oncogenesis and chemoresistance activity of tumor cells. miR-203 has a critical role in sustaining the proliferation and self-renewal of LSCs through targeting the 3′-UTR regions of survivin and BMI-1. Therefore, the miR-203/survivin/BMI-1 axis could be considered a therapeutic target and prognosis/diagnostic marker for treating leukemia.^250^ A recent study investigated the expression profile of miR-193 b-5p/3p in 161 pediatric and 187 adult AML patients. It is found that this miRNA was down-regulated in cytogenetical subgroups of patients and introduced as an independent poor prognostic marker in pediatric AML. It also studied the tumor-suppressive effect of miR-193 b in patient-derived xenografts, human AML blasts, and miR-193 b knockout mice, and revealed that this miRNA regulated the self–renewal properties of HSCs and determined the progression of AML. Therefore, miR-193 b is an endogenous tumor suppressor with warning potential to better selection of HSC transplantation candidates. miR-193 b probably through targeting the MAPK signaling cascade and the key cell cycle regulatory protein cyclinD1 (CCND1), induces apoptosis and blocks the G1/S-phase in AML.^251^ High expression of miR-126 was found in LSCs and leukemic progenitors (LPs) of AML patients and cell lines. This miRNA down-regulated tumor necrosis factor receptor-associated factor 7 (TRAF7) and subsequently blocked the c-FLIP pathway, inducing an anti-apoptotic effect on AML cell lines.^252^ Furthermore, miR-34c-5p regulated multiple signaling pathways involved in senescence. It has been shown that this miRNA is down-regulated in AML stem cells and this lower expression is closely associated with poor prognosis and responses to therapy in AML patients. In addition, an experimental study showed that restoring miR-34c-5p expression could prevent leukemia development and promote LSC senescence via interaction with the p53-p21Cip1-Cyclin-dependent kinase (CDK)/Cyclin or p53-independent CDK/Cyclin pathways. Therefore, this miRNA may be a novel antileukemic biomarker to reinitiate the senescence of LSCs.^253^ miR-339-5p has opportunities to target anti-leukemic strategies. Overexpression of FGFR1 kinases promoted stem cell phenotype and resistance to apoptosis, and consequently induced stem cell leukemia/lymphoma syndrome (SCLL). Indeed, FGFR1 kinase through regulating miRNA expression, importantly miR-339-5p, enhanced the survival and proliferation of LSCs. Inhibition of miR-339 could diminish the viability of LSCs by targeting the BCL2L11 and BAX pro-apoptotic genes.^254^ It has been demonstrated that hsa-miR-181a-5p is a prognostic marker in AML patients treated with intensive induction chemotherapy and autologous stem cell transplant.^255^

## Conclusion

Current cancer treatment strategies focus on inhibiting cancer-initiating cells as pivotal players in the propagation and relapse of tumors. Therefore, targeting signaling pathways associated with the stemness properties of these cells is gaining even more importance in this viewpoint. However, further research is required to determine specific markers of LSCs, identify specific targets, and increase effective LSC-based management of leukemia.

## Author contributions

M. F., SH. A., A. N., S. N., M. SH., F. ND., O. A., SE. KH., and SH. U. contributed to manuscript writing. All authors approved the submitted version of the article and agreed to be personally accountable for the authors' contributions and to ensure the accuracy or integrity of any part of the work. All authors read and approved the final manuscript.

## Data availability

The datasets used and/or analyzed during the current study are available from the corresponding author upon reasonable request.

## Conflict of interests

The authors declare that there are no competing interests.
